# Investigation of Gambling Behavior, Self-Confidence and Psychological Resilience Levels of University Students

**DOI:** 10.1007/s10899-024-10317-3

**Published:** 2024-05-28

**Authors:** Songul Duran, Özlem Demirci, Filiz Akgenç

**Affiliations:** 1 Health Services Vocational College, Care of Elderly Program, İzmir Demokrasi University, İzmir, Türkiye; 2 Kartal Kosuyolu High Specialization Training and Research Hospital Cardiology Clinic, İstanbul, Türkiye; 3 Faculty of Health Sciences, Kocaeli University, Kocaeli, Türkiye

**Keywords:** Addiction, Gambling, Self- confidence, Psychological resilience, University student

## Abstract

The aim of this study is to examine the relationship between gambling behavior, self-confidence, and psychological resilience levels among university students. Additionally, the study aims to investigate the relationship between gambling behavior and socio-demographic variables. This descriptive and cross-sectional study was conducted between April and June 2023. The research employed a questionnaire, the Brief Psychological Resilience Scale, the South Oaks Gambling Screen (SOGS), and the Self-Confidence Scale. The study was carried out online, reaching 229 students through Google Forms. According to the SOGS scores, 4.8% of the students are at risk of gambling addiction. Male students have statistically significantly higher SOGS scores than female students. The SOGS score is significantly higher in working students, smokers, and alcohol drinkers (*p* < 0.05). No statistically significant relationship was found between the students’ SOGS scores and the self- confidence scale (*p* = 0.637) and the brief resilience scale (*p* = 0.675). It is thought that training should be given to risky groups in order to prevent gambling behavior. In addition, it is thought that supporting university students to be active in different arts and sports fields may have a positive effect on preventing and reducing addictions.

## Introduction

Gambling addiction has become a serious public health issue in recent years. In the Diagnostic and Statistical Manual of Mental Disorders (DSM-5), only ‘gambling disorder’ is included under the non-drug-related disorder category (Genovese et al., 2022). Gambling disorder (GD) was removed from the impulse control disorder category in DSM-5, reclassified as an addictive disorder, and included in the behavioral addiction category. In line with this reclassification, Gambling Disorder (GD) includes criteria related to tolerance, where individuals engage in increasingly higher levels of gambling over time to attain the desired outcome, as well as withdrawal symptoms, manifesting as irritability when attempting to abruptly cease gambling (Potenza et al., 2019). The World Health Organization’s 11th and latest edition of the International Classification of Diseases (ICD-11) has addressed that GD has significant etiological, clinical, and neurological similarities with substance use disorders, and therefore it would be more appropriate to define GD as an addictive disorder rather than an impulse control disorder (Kim & Hodgins, 2019). For most gamblers, gambling is merely a form of entertainment. Losing at gambling doesn’t constitute a significant disaster for them, and they move on to other activities. However, the allure of gambling is much stronger for certain young adults. Individuals begin to gamble more than they initially intend to and start losing more than they can afford while playing. Their inability to control their behavior is an indication of gambling addiction problem (Evangelista et al., 2021). One of the pioneering tools developed specifically to screen for gambling problems is the South Oaks Gambling Screen, which is based on the operational definition of pathological gambling in the DSM-III edition and is often administered as a self-report tool (Granero et al., 2020). The Problem Gambling Severity Index (PGSI) is a scale used to determine the total gambling severity score, categorized into four levels of severity ranging from non-problem gambling to problem gambling. PGSI, compared to DSM and other population-level tools, shows higher accuracy in identifying individuals at risk of developing gambling issues. PGSI demonstrates greater accuracy compared to DSM and other population-level tools. It excels in identifying individuals at risk of developing gambling issues (Butler & Strouse, 2022). In this research, PGSI was used as a measurement tool.

Adolescents are among the at-risk groups for pathological gambling addiction (Çakıcı et al., 2019). It is also estimated that the prevalence of gambling problems among adolescents is two to four times higher than among adults (Pisarska & Ostaszewski, 2020). Epidemiological studies estimate that the prevalence of pathological gambling among adults ranges from 1.1 to 3.5% (Moreira et al., 2024). Although gambling is not legal in Türkiye, it is understood that online gambling is spreading rapidly with developing technology and increasing internet usage (Vayısoğlu et al., 2019). Gambling in Türkiye is prohibited by legal regulations. Betting games are free. Therefore, legal and criminal sanctions are applied for gambling. Betting games are officially increasing their activities day by day (Akça, 2019). A study conducted in Turkish society reveals that betting games are not considered as negative as gambling (Bayındır, 2018). It is estimated that between 1.9% and 15% of adolescents engage in gambling, and approximately 28% of these may be at risk of developing problematic gambling behavior (Bozzato et al., 2020). In a meta-analysis study, the estimated rate of potential pathological gamblers among students was calculated as 6.13%, and the rate of problematic gambling was calculated as 10.23% (Nowak, 2018). University students demonstrate a higher sensitivity to exposure to gambling and similar risky behaviors, which consequently increases their rate of gambling and gambling addiction (Grande-Gosende et al., 2020). Increasing independence from family and the desire to fill free time increases students’ tendency to gamble (Wong et al., 2022). It has been noted that university students are exposed to gambling, as well as alcohol and drug use, on campuses and within university environments (Kapukotuwa et al., 2023). University years can be characterized by many problems such as the chaotic period of adolescence, separation from home and family, adapting to a new environment and uncertainty about the future. For this reason, tobacco, alcohol and substance use among university students may increase due to excessive anxiety and stress brought about by these problems (Aslan et al., 2021). Experiencing the excitement of gambling, living in a social community that exhibits and encourages gambling behavior, trying to cope with and escape from intensely felt negative emotions such as stress, shame, anger, guilt, hopelessness can be the primary sources of motivation for individuals (Çelik et al., 2022). It has been reported that university students with gambling addiction experience a significant decline in their academic performance, engage in socially isolating behaviors, face difficulties in social relationships, and are at a high risk of suicidal thoughts and attempts (Richard et al., 2019). Therefore, it is important to identify the factors contributing to the etiology of addiction and to take necessary measures accordingly. Among the risk factors for gambling are high income level, being male, and being younger in age (Grönroos et al., 2022; Lind et al., 2022). In a study, parental gambling behavior and maternal education level were associated with regular gambling in both genders (Hollén et al., 2020). Dependents are notable for their very limited or nonexistent social lives and their very low or nonexistent self-confidence (Toker & Baturay, 2016). It is known that lack of self-confidence is among the triggers for substance addiction (Tonkuş et al., 2022).

Pathological gamblers have been found to have lower self-esteem and mood disorders and engage in risk-taking behaviors to increase their sense of excitement and physiological arousal (Choi & Kim, 2021). Individuals who engage in gambling at casinos or participate in illegal gambling exhibit a greater tendency to seek excitement (Raylu & Oei, 2002). It was determined that the variable that best predicted internet addiction was lack of social self-confidence. (İskender, 2018). Psychological resilience is also defined as having developable attitudes and skills that can overcome current difficulties, facilitate the development of a better attitude than expected, and enable individuals to endure despite stressful life experiences and negative and difficult living conditions (Çelik et al., 2022). Concepts such as adaptive coping strategies, social support, spirituality or religious involvement, interpersonal skills or competence, personal autonomy or self-efficacy have been discussed as negatively correlated with gambling addiction issues (Dowling et al., 2021). Individuals with psychological resilience are capable of dealing with stressful situations and adapting by utilizing functional coping strategies. There is evidence that psychological resilience can protect individuals, especially young people, from gambling-related problems (Sirola et al., 2023). It is emphasized that individuals with high psychological resilience are more likely to access social resources that improve psychological health, act appropriately to achieve a healthy lifestyle, process cognitive and sensory data, and use existing resources correctly (Kocabıyık & Bacıoğlu, 2022).

Numerous studies indicate that pathological gambling addiction can lead to both individual and societal issues. Therefore, this study aims to determine university students’ gambling behavior, self-confidence, and psychological resilience levels, and to establish the relationship between gambling behavior and levels of self-confidence and psychological resilience. Additionally, the study aims to identify socio-demographic factors influencing gambling behavior. The reason for targeting university students as the study population is their presence in the at-risk group for gambling behavior. In conclusion, the objectives of this study were as follows: (I) to determine gambling behavior, self-confidence, and psychological resilience scale scores among students; (II) to establish the relationship between scales and socio-demographic characteristics; and (III) to analyze the correlations between the scales.

## Method

The sample of the research consists of university students who are studying at a University between the months of April and June 2023. The population of the study includes university students aged 18 and above, willing to participate in the study, and enrolled at a University. By employing simple random sampling among the faculties of the university, two faculties were included in the sample. The total number of students in these two faculties was found to be 9371. The sample size was determined to be 229 through sample calculation with known universe. Surveys were sent to 250 randomly selected students to reach the sample, and the study was terminated when we received 229 complete surveys. Ethical approval was obtained from the İzmir Demokrasi University Research and Ethics Board (Number:2023/04 − 03 Date: 29.03.2023). The forms were delivered to the students via their WhatsApp and email accounts. A written explanation about the study was included at the beginning of the questionnaire.

## Measures

In the study, a questionnaire prepared by the researcher, Brief Psychological Resilience Scale, South Oaks Gambling Screening Test (SOGS) and Self-Confidence Scale were used. The questionnaire form includes questions about the socio-demographic characteristics of the participants.

### Brief Psychological Resilience Scale (BPRS)

BPRS, developed by Smith et al. (2008) (Smith et al., 2008), was adapted into Turkish by Doğan in 2015. It is a scale that aims to evaluate the capacity of individuals to recuperate, return to their previous functionality, recover and adapt, and consists of a total of 6 items. The scale is in a 5-point Likert style. 1: Not at all appropriate 2: Not appropriate 3: Somewhat appropriate 4: Appropriate 5: Completely appropriate. Items 2, 4, and 6 are reverse coded. As a result of exploratory and confirmatory factor analyses, the scale has a single-factor structure. The Kaiser-Meyer Olkin (KMO) coefficient of the scale is 0.85, and the Bartlett’s Test of Sphericity χ2 value is 594.955 (*p* < 0.001), indicating that the scale has a high level of reliability (Doğan, 2015).

### South Oaks Gambling Screen (SOGS)

The Turkish reliability and validity study of the SOGS (17), developed by Lesieur and Blume in 1987, was conducted by Duvarcı and Varan in the year 2001. The scale is one-dimensional and consists of 19 items. While 17 of the 20 items included in the scoring of the SOGS were seen to distinguish those with pathological gambling problems from those without, three items that were determined to be ineffective were removed from the scale, and two new items specific to our culture were added instead (Three items deemed culturally inappropriate for Turkish culture; If you borrowed money to gamble or to pay gambling debts, who or where did you borrow from? from your spouse, you cashed in stocks, bonds or other securities you borrowed on your checking account (passed bad checks). As a result of the conducted analyses, the Turkish version of the SOGS has taken the form of a 19-item scale with a cutoff point of 8 points. The internal consistency coefficient of the nineteen-item SOGS was Cronbach’s alpha = 0.8772 and the test-retest correlation was *r* = 0.95. In the study, which was adapted to the Turkish sample, it was stated that it would be appropriate to classify those who score 8 or higher out of a total of 19 points as likely pathological gamblers (Duvarcı & Varan, 2001).

### Self-Confidence Scale

The Self-Confidence Scale was developed by Akın in the year 2007. It consists of a total of 2 sub-dimensions (inner self confidence and externalself confidence) and 33 items. The scale is prepared in a 5-point Likert-type format, with the highest possible score of 165 and the lowest score of 33. (1) Never, (2) Rarely, (3) Often, (4) Generally, and (5) Always represent the response options. Structural and coherent validity studies were conducted as scale validity studies, internal consistency and test-retest reliability and item analysis were performed as reliability studies. The internal consistency coefficients of the Self-Confidence Scale were found to be 0.83 for the whole scale, 0.83 and 0.85 for the internal self-confidence and external self-confidence subscales, respectively. A high score from the scale indicates a high level of self-confidence (Akın, 2007).

### Statistical Analysis

The data were evaluated in computer using SPSS 25 (Statistical Package for Social Sciences) package program and the level of significance was accepted as *p* < 0.05. The conformity of the data to the normal distribution was evaluated with the Shapiro-Wilk and Kolmogorov-Smirnov tests, and percentages and averages were used in the evaluation of the data, and the student t test/ ANOVA and Tukey test were used for the intergroup comparisons. Correlation analysis was used to evaluate the relationship between the scales.

### Ethical Condition

In order to carry out the study, permission was obtained from the Scientific Research and Publication Ethics Committee of İzmir Demokrasi University (Date: 29.03.2023, number: 2023/04 − 03). Institutional permission was obtained from the institution where the study was conducted. Volunteering criteria were taken as basis for participation in the research. All students participating in the study were informed and their voluntary consent was obtained.

## Findings

### Socio-Demographic Characteristics of Students

50.2% of the participants are female and 50.7% are studying at the Faculty of Engineering. 81.2% of the students are not working and the family income of 81.2% is sufficient to cover their expenses. 154 students (67.2%) stated that they lived in the province, education level of the mothers of 46.7% is primary school or below, and the education level of the fathers of 30.6% is primary school or below. 1.7% use drugs, 31.4% use alcohol and 28.4% smoke (Table 1).

**Table 1:** Socio-demographic characteristics of the participants

Sociodemographic features	*n*	%
**Gender**		
Male	114	49.8
Female	115	50.2
**Faculties**		
Faculty of Engineering	113	49.3
Faculty of Health Science	116	50.7
**Working status**		
Yes	43	18.8
No	186	81.2
**Place of residence**		
Town/District	75	32.8
Province center	154	67.2
**Mother’s level of education**		
Primary school graduate and below	107	46.7
Secondary school	29	12.7
High school graduate	60	26.2
Bachelor’s degree and above	33	14.4
**Father’s level of education**		
Primary school graduate and below	70	30.6
Secondary school	42	18.3
High school graduate	57	24.9
Bachelor’s degree and above	60	26.2
**Smoking**		
Yes	65	28.4
No	164	71.6
**Alcohol**		
Yes	72	31.4
No	157	68.6
**Substance use**		
Yes	4	1.7
No	225	98.3
**Potentially pathological gamblers**		
Yes	11	4.8
No	218	95.2

### Mean Scores of Students from Scales

In the study, the average score of the students from the SOGS was 0.93 ± 2.47 and according to the cut-off point of the scale, 4.8% of them had a risk of gambling addiction. The mean score of the self-confidence scale is 118.72 ± 20.84, the mean score of internal self-confidence, which is one of the sub-dimensions of the scale, is 61.35 ± 11.12, and the mean score of external self-confidence is 57.43 ± 11.00. The mean score of the psychological resilience scale is 18.20 ± 4.93 (Table 2).

**Table 2:** Descriptive statistics of students’ gambling, self-confidence and resilience (*n* = 229)

Scales	Mean ± SD
**South Oaks Gambling Screening Test (SOGS)**	0.93 ± 2.47
**Self-Confidence**	118.66 ± 20.75
Internal self-confidence	61.35 ± 11.12
External self-confidence	57.41 ± 10.986.49
**Brief Resilience Scale**	18.20 ± 4.93
Abbreviation: SD, standard deviation	

### Comparison of Students’ Scores from the SOGS Level According to their Socio-Demographic Characteristics

Table 3 shows the distribution of students’ scores from the SOGS in terms of some variables. In the study, male students’ (1.58 ± 3.24) SOGS scores were higher than female students (0.28 ± 1.0).

**Table 3:** Students’ characteristics and comparison of gambling

Characteristics	Mean ± SD	t / F	*p*
**Gender** Female Male	0.28 ± 1.0 1.58 ± 3.24	-4.105	**0.000** ^**^
**Working status** Yes No	1.67 ± 3.63 0.76 ± 2.10	2.189	**0.030** ^*^
**Smoking** Yes No	2.18 ± 3.97 0.43 ± 1.23	5.055	**0.000** ^**^
**Alcohol** Yes No	1.94 ± 3.57 0.47 ± 1.57	-4.333	**0.000** ^**^
**Substance use** Yes No	3.0 ± 6.0 0.89 ± 2.38	1.687	0.093

^*^p < 0.05, ^**^p < 0.01

The SOGS score of smokers (2.18 ± 3.97) was statistically significantly higher compared to non-smokers (0.43 ± 1.23), and the SOGS score of alcohol consumers (1.94 ± 3.57) was also significantly higher compared to non-consumers (0.47 ± 1.57) (*p* < 0.05). The SOGS score of the working students (1.67 ± 3.63) compared to the non-working students (0.76 ± 2.10) was statistically significantly higher (*p* < 0.05).

There was no statistically significant difference in the scores obtained from the South scale according to the education level of the mother, the education level of the father and the substance use status of the students.

### Correlations of Students’ SOGS, Self-Confidence Scale, and Brief Resilience Scale Scores

Table 4 shows the correlation analysis among students’ SOGS, Self-Confidence Scale, and Brief Resilience Scale scores. No statistically significant relationship was found between the students’ SOGS scores and the self-confidence scale (*p* = 0.637) and the brief resilience scale (*p* = 0.675).

**Table 4:** Correlations of Students’ SOGS, Self-Confidence Scale, and Brief Resilience Scale Scores

Variables		Self-confidence	Brief Resilience Scale
**SOGS score for non-pathological gamblers**	**r**	0.116	0.54
	**p**	0.087	0.425
**SOGS score for potential pathological gamblers**	**r**	-0.161	-0.143
	**p**	0.637	0.675

## Discussion

In this study, the relationship between self confidence and resilience levels, socio-demographic characteristics affecting gambling behavior, and the relationship between gambling behavior and self-confidence and resilience were examined. In the study, according to the cut-off score of the scale, 4.8% were found to be in the group of those who gambled at a probable pathological level. Aslan et al. determined the potential pathological gambling behavior in university students at the level of 2% (Aslan et al., 2021). Koç et al. detected a possible pathological level of gambling addiction in 14.2% of university students (Koç et al., 2023). Bozzato et al. have identified risky gambling behavior in 8.8% of adolescents and classified 3.8% as problematic players (Bozzato et al., 2020). Çelik et al. determined the probable pathological gambling addiction in university students at the level of 1.2% (Çelik et al., 2022). In their study, Choi and Kim found that 16.3% of the participants were in the low-risk, 9.4% in the medium-risk and 5.4% in the problematic group in terms of problematic gambling behavior (Choi & Kim, 2021). Yokomitsu et al. found the rate of probable problem gambling among students to be 5.1%, close to our study (Yokomitsu et al., 2019). In a study conducted with Spanish adolescents, it was found that 20.6% of the participating adolescents had engaged in gambling in the last 12 months (Pérez-Albéniz et al., 2022). The discrepancy between the results of our study and those of studies conducted in our country may be attributed to the different characteristics of the sample groups. For instance, in our study, the ratio of male to female students is similar. However, in some studies, a higher proportion of female participants may have led to a lower incidence of gambling addiction. Similarly, it is thought that other socio-demographic characteristics (income level, employment status, etc.) may be associated with different results.

In the literature, it has been stated that gambling behavior is more common in men than in female (Ciccarelli et al., 2020; Mond et al., 2019; Sirola et al., 2023). In this study, the SOGS score of male students was found to be higher than that of female students. Similarly, Koç and colleagues also found higher SOGS scores in males compared to females (Koç et al., 2023). Choi and Kim similarly found a higher SGOS score in men than in female (Choi & Kim, 2021). Cakici et al. found pathological gambling behavior at a higher rate in male students than in females (Çakıcı et al., 2020). The higher prevalence of gambling behavior in men is likely due to the fact that men tend to take higher risks and exhibit more impulsivity compared to female (Ciccarelli et al., 2020).

Considering the results of the study, the SOGS score of the working students (1.67 ± 3.63) was significantly higher than the non-working students (0.76 ± 2.10) (*p* < 0.05). In another study, it was stated that unemployed participants were more at risk of having gambling problems (Evangelista et al., 2021). Şen and Pirinci, in their study, found that middle-income students showed more problematic gambling behavior compared to high-income students (Şen & Pirincci, 2023). Koç et al. determined that gambling behavior differs according to income level (Koç et al., 2023). Providing encouraging training in various arts and sports activities instead of gambling behavior may be beneficial for these students. Another point we need to consider here is that no single socio-demographic characteristic alone will pose a risk for gambling, and multiple factors may play a role in this. It is suggested that preventive counseling be provided to at-risk groups by considering various factors such as individuals’ genetic predisposition, coping styles, personality traits, resilience, and similar characteristics, all of which together contribute to the development of gambling addiction.

In this study, SOGS scores of smokers and alcohol consumers were found to be significantly higher than non-smokers and non-alcohol consumer. Alcohol and other addictions have been reported to be associated with gambling behavior (Evangelista et al., 2021). Similarly, Koç et al. found a significantly higher SOGS score in students who smoke and drink alcohol (Koç et al., 2023). Pisarska et al. (2020) found a positive relationship between gambling behavior and substance addiction in their study (Pisarska & Ostaszewski, 2020). Şen and Pirincci determined that students who drink alcohol have a higher level of gambling behavior compared to those who do not (Şen & Pirincci, 2023). It is believed that factors playing a role in susceptibility to addiction may be common, and individuals exhibiting risky behaviors are at risk for other types of addiction as well. These individuals should be observed and supported.

In this study, no statistically significant relationship was found between problematic gambling behavior and self-confidence and resilience. Considering that there is a connection between stressful life events and gambling, it is thought that psychological resilience may be protective for gambling behavior. It was determined that having more confidence in the ability to stay away from gambling is among the facilitating factors in treating gambling addiction (Gomes & Pascual-Leone, 2009). Sirola et al. (2023) did not find a significant relationship between resilience and gambling behavior in their study (Sirola et al., 2023). Mishra et al. identified resilience as a protective factor for pathological gambling (Mishra et al., 2019). Çelik et al. did not find a significant relationship between pathological gambling behavior and psychological resilience (Çelik et al., 2022). Although there are findings in the literature that psychological resilience may be protective for gambling behavior, there are also studies that determined that there is no relationship between them. Therefore, it is recommended to conduct studies in a larger population. Additionally, individuals with low levels of self-confidence should also be supported, as this is important in addressing the issue.

## Conclusion

In this study, 4.8% of the students are in the risk group for pathological gambling. In addition, the SOGS score was found to be higher in men, workers, and smokers and alcohol smokers. It is recommended to conduct supportive and preventive training programs for the risky group. In addition, conducting studies with a large sample and examining the relationships with different variables may benefit the literature.

## Data Availability

The data that support the findings of this study are available from the corresponding author upon request.

## References

[CR1] Akça, Y. (2019). Sanal Bahis Siteleri Mi Ya Da Sanal Kumar Siteleri Mi? *Business & Management Studies: An IIternational Journal*, *4*, 1446–1466.

[CR2] Akın, A. (2007). Öz-Güven ölçeğini’nin geliştirilme ve psikometrik özellikleri. *Abant İzzet Baysal Üniversitesi Eğitim Fakültesi Dergisi*, *7*(2), 167–176.

[CR3] Aslan, E. A., Batmaz, S., Çelikbas, Z., Kilinçel, O., Sayar, G. H., & Ünübol, H. (2021). Prevalence of risk for substance-related and behavioral addictions among university students in Turkey. *Addicta: The Turkish Journal on Addictions*, *8*(1), 35–44. 10.5152/ADDICTA.2021.21023

[CR4] Bayındır, G. (2018). Kumar ve Şans Oyunarına Toplumsal Bakış: Niğde Örneği. *Milli Kültür Araştırmaları Dergisi*, *2*(2), 58–83.

[CR5] Bozzato, P., Longobardi, C., & Fabris, M. A. (2020). Problematic gambling behaviour in adolescents: Prevalence and its relation to social, self-regulatory, and academic self-efficacy. *International Journal of Adolescence and Youth*, *25*(1), 907–919. 10.1080/02673843.2020.1772842

[CR6] Butler, A. M., & Strouse, S. M. (2022). An Integrative Review of Incivility in nursing education. *Journal of Nursing Education*, *61*(4), 173–178. 10.3928/01484834-20220209-01 35384766 10.3928/01484834-20220209-01

[CR7] Çakıcı, M., Çakıcı, E., Karaaziz, M., & Babayiği, A. (2019). A review of Problem and pathological gambling in North Cyprus. *Cyprus Turkish Journal of Psychiatry and Psychology*, *1*(2), 123–128. 10.35365/ctjpp.19.1.15

[CR8] Çakıcı, M., Fırat, Y., Babayiğit, A., & Karaaziz, M. (2020). The comparison between the sociodemographic characteristics and the problematic internet use between gamblers and non-gamblers among university students. *Klinik Psikiyatri Dergisi*, *23*(1), 34–42. 10.5505/kpd.2020.93685

[CR9] Çelik, S., Öztürk, A., Kes, E., & Kurt, A. (2022). The relationship of gambling with sensation-seeking behavior and psychological resilience in university students. *Perspectives in Psychiatric Care*, *58*(4), 2199–2207. 10.1111/ppc.13047 35133668 10.1111/ppc.13047

[CR10] Choi, J., & Kim, K. (2021). The relationship between impulsiveness, self-esteem, irrational gambling belief and problem gambling moderating effects of gender. *International Journal of Environmental Research and Public Health*, *18*(10), 5180. 10.3390/ijerph18105180 34068198 10.3390/ijerph18105180 PMC8153021

[CR11] Ciccarelli, M., Nigro, G., Griffiths, M. D., D’Olimpio, F., & Cosenza, M. (2020). The associations between maladaptive personality traits, craving, Alcohol Use, and adolescent Problem Gambling: An Italian survey study. *Journal of Gambling Studies*, *36*(1), 243–258. 10.1007/s10899-019-09872-x 31300930 10.1007/s10899-019-09872-x

[CR12] Doğan, T. (2015). Kısa Psikolojik Sağlamlık Ölçeği’nin Türkçe uyarlaması: Geçerlik ve güvenirlik çalışması adaptation of the brief resilience scale into Turkish: A validity and reliability study. *The Journal of Happiness & Well-Being*, *3*(1), 93–102.

[CR13] Dowling, N. A., Aarsman, S. R., & Merkouris, S. S. (2021). Risk, compensatory, and protective factors in problem gambling: The role of positive mental health characteristics. *Addictive Behaviors*, *112*(August 2020), 106604. 10.1016/j.addbeh.2020.106604 10.1016/j.addbeh.2020.106604 32805541

[CR14] Duvarcı, İ., & Varan, A. (2001). South oaks Kumar Tarama Testi Türkçe Formu Güvenirlik ve Geçerlik Çalışması. *Türk Psikiyatri Dergisi*, *12*(1), 34–45.

[CR15] Evangelista, L. D., Lagumbay, R. C. S., & Pagcaliwagan, K. D. (2021). Gambling behavior of young adults: A basis for an intervention program. *Interdisciplinary Journal of Apllied and Basic Subjects*, *1*(7), 36–44.

[CR16] Genovese, C., Spataro, P., Venuto, R., La Fauci, V., Cosenza, B., Palamara, M. A. R., Mazzù, F., Campanella, G., Cipriano, G., Pantaleo, L., Fedele, F., Di Pietro, A., & Squeri, R. (2022). Gambling addiction in a Cohort of University Students of Southern Italy: A Cross Sectional Study. *EuroMediterranean Biomedical Journal*, *17*(25), 117–122. 10.3269/1970-5492.2022.17.25

[CR17] Gomes, K., & Pascual-Leone, A. (2009). Primed for change: Facilitating factors in problem gambling treatment. *Journal of Gambling Studies*, *25*(1), 1–17. 10.1007/s10899-008-9111-y 19015963 10.1007/s10899-008-9111-y

[CR18] Grande-Gosende, A., López-Núñez, C., García-Fernández, G., Derevensky, J., & Fernández-Hermida, J. R. (2020). Systematic Review of Preventive Programs for reducing Problem Gambling behaviors among Young adults. *Journal of Gambling Studies*, *36*(1), 1–22. 10.1007/s10899-019-09866-9 31168687 10.1007/s10899-019-09866-9

[CR19] Granero, R., Jiménez-Murcia, S., Fernández-Aranda, F., Pino-Gutiérrez, D., Mena-Moreno, A., Mestre-Bach, T., Gómez-Peña, G., Moragas, M., Aymamí, L., Giroux, N., Grall-Bronnec, I., Sauvaget, M., Codina, A., Vintró-Alcaraz, E., Lozano-Madrid, C., Camozzi, M., Agüera, M., Sánchez-González, Z., Casalé-Salayet, J., & Menchón, G., J. M (2020). Presence of problematic and disordered gambling in older age and validation of the South oaks Gambling Scale. *Plos One*, *15*(5), e0233222. 10.1371/journal.pone.0233222 32428026 10.1371/journal.pone.0233222 PMC7237015

[CR20] Grönroos, T., Kouvonen, A., Kontto, J., & Salonen, A. H. (2022). Socio-demographic factors, gambling Behaviour, and the level of Gambling expenditure: A Population-based study. *Journal of Gambling Studies*, *38*(4), 1093–1109. 10.1007/s10899-021-10075-6 34606033 10.1007/s10899-021-10075-6 PMC9653360

[CR22] Hollén, L., Dörner, R., Griffiths, M. D., & Emond, A. (2020). Gambling in young adults aged 17–24 years: A Population-based study. *Journal of Gambling Studies*, *36*(3), 747–766. 10.1007/s10899-020-09948-z 32306233 10.1007/s10899-020-09948-z PMC7395026

[CR23] İskender, M. (2018). Investigation of the effects of Social Self-Confidence, Social Loneliness and Family Emotional loneliness variables on internet addiction. *Malaysian Online Journal of Educational Technology*, *6*(3), 1–10. 10.17220/mojet.2018.03.001

[CR24] Kapukotuwa, S., Bonsu, L., Chatterjee, A., Fudolig, M., & Sharma, M. (2023). Examining the Gambling Behavior of University students: A cross-sectional Survey applying the Multi-theory Model (MTM) of Health Behavior Change in a single Institution. *Healthcare (Switzerland)*, *11*(15). 10.3390/healthcare11152151 10.3390/healthcare11152151 PMC10419051 37570391

[CR25] Kim, H. S., & Hodgins, D. C. (2019). A review of the evidence for considering Gambling Disorder (and other behavioral addictions) as a disorder due to addictive behaviors in the ICD-11: A focus on case-control studies. *Current Addiction Reports*, *6*(3), 273–295. 10.1007/s40429-019-00256-0

[CR26] Koç, Ş., Kocakaya, R., Türkmen, A., & Çakıcı, A. (2023). University Students ’ Gaming and Gambling Behaviors. *Journal of Gambling Studies*, *Advance on*. 10.1007/s10899-023-10209-y 37115422

[CR27] Kocabıyık, O., & Bacıoğlu, S. (2022). Predictive roles of psychological resilience and coping skills on Social Media Addiction. *Psikiyatride Güncel Yaklaşımlar*, *14*(Suppl. 1), 137–146. 10.18863/pgy.1137812

[CR29] Lind, K., Marionneau, V., Järvinen-Tassopoulos, J., & Salonen, A. H. (2022). Socio-Demographics, Gambling Participation, Gambling Settings, and addictive behaviors Associated with Gambling modes: A Population-based study. *Journal of Gambling Studies*, *38*(4), 1111–1126. 10.1007/s10899-021-10074-7 34623554 10.1007/s10899-021-10074-7 PMC9653367

[CR30] Mishra, S., Beshai, S., Wuth, A., & Refaie, N. (2019). Risk and protective factors in problem gambling: An examination of psychological resilience. *International Gambling Studies*, *19*(2), 241–264. 10.1080/14459795.2018.1545242

[CR31] Mond, J., Skromanis, S., Purton, T., Cooling, N., Fan, F., Harris, K., Bridgman, H., Presser, J., & Rodgers, B. (2019). Gambling behaviour, problem gambling and reasons for gambling among international students in Tasmania, Australia. *Journal of Gambling Studies*, *35*(1), 155–170. 10.1007/s10899-018-09819-8 30617902 10.1007/s10899-018-09819-8

[CR32] Moreira, D., Dias, P., Azeredo, A., Rodrigues, A., & Leite, Â. (2024). A systematic review on intervention treatment in pathological gambling. *International Journal of Environmental Research and Public Health*, *21*(3), 346. 10.3390/ijerph21030346 38541345 10.3390/ijerph21030346 PMC10970379

[CR33] Nowak, D. E. (2018). A Meta-analytical synthesis and examination of pathological and Problem Gambling Rates and Associated moderators among College Students, 1987–2016. *Journal of Gambling Studies*, *34*(2), 465–498. 10.1007/s10899-017-9726-y 29058167 10.1007/s10899-017-9726-y

[CR34] Pérez-Albéniz, A., Gil, M., Díez-Gómez, A., Martín-Seoane, G., & Lucas-Molina, B. (2022). Gambling in Spanish adolescents: Prevalence and association with mental health indicators. *International Journal of Environmental Research and Public Health*, *19*(1), 129. 10.3390/ijerph19010129 10.3390/ijerph19010129 PMC8750538 35010388

[CR35] Pisarska, A., & Ostaszewski, K. (2020). Factors associated with youth gambling: Longitudinal study among high school students. *Public Health*, *184*, 33–40. 10.1016/j.puhe.2020.03.017 32620298 10.1016/j.puhe.2020.03.017

[CR36] Potenza, M. N., Balodis, I. M., Derevensky, J., Grant, J. E., Petry, N. M., Verdejo-Garcia, A., & Yip, S. W. (2019). Gambling disorder. *Nature Reviews Disease Primers*, *5*(1). 10.1038/s41572-019-0099-7 10.1038/s41572-019-0099-7 31346179

[CR37] Raylu, N., & Oei, T. P. S. (2002). Pathological gambling: A comprehensive review. *Clinical Psychology Review*, *22*(7), 1009–1061. 10.1016/S0272-7358(02)00101-0 12238245 10.1016/s0272-7358(02)00101-0

[CR38] Richard, J., Paskus, T. S., & Derevensky, J. L. (2019). Trends in gambling behavior among college student-athletes: A comparison of 2004, 2008, 2012 and 2016 NCAA survey data. *Journal of Gambling Issues*, *41*(May), 73–100. 10.4309/jgi.2019.41.5

[CR39] Şen, M. A., & Pirincci, E. (2023). Examination of hookah usage and gambling status of university students*. *Journal of Substance Use*, *00*(00), 1–8. 10.1080/14659891.2023.2238310

[CR40] Sirola, A., Nyrhinen, J., & Wilska, T. A. (2023). Psychosocial perspective on Problem Gambling: The role of Social relationships, Resilience, and COVID-19 worry. *Journal of Gambling Studies*, *39*, 1467–1485. 10.1007/s10899-022-10185-9 36622471 10.1007/s10899-022-10185-9 PMC9827443

[CR41] Smith, B. W., Dalen, J., Wiggins, K., Tooley, E., Christopher, P., & Bernard, J. (2008). The brief resilience scale: Assessing the ability to bounce back. *International Journal of Behavioral Medicine*, *15*(3), 194–200. 10.1080/10705500802222972 18696313 10.1080/10705500802222972

[CR42] Toker, S., & Baturay, M. H. (2016). Antecedents and consequences of game addiction. *Computers in Human Behavior*, *55*, 668–679. 10.1016/j.chb.2015.10.002

[CR43] Tonkuş, M., Elveren, A., & Tokmak, Ş. (2022). Bağımlı Bireylerin Yaşadıkları Ruhsal Sorunlar ve Önlemeye Yönelik Uygulamalar. *Journal of Medical Sceineces*, *3*(4), 206–213.

[CR44] Vayısoğlu, S. K., Öncü, E., & Güven, Y. (2019). The frequency of Gambling among University students and its relationships to their sensation-seeking Behaviors*. *Addicta: The Turkish Journal on Addictions*, *6*(1), 69–90. 10.15805/addicta.2019.6.1.0041

[CR45] Wong, I. L. K., So, E. M. T., & Chu, C. H. (2022). Gambling Behavior among Hong Kong College and University students. *International Journal of Mental Health and Addiction*, *20*(4), 2265–2276. 10.1007/s11469-021-00512-3

[CR46] Yokomitsu, K., Sakai, T., Irie, T., Tayama, J., Furukawa, H., Himachi, M., Kanazawa, J., Koda, M., Kunisato, Y., Matsuoka, H., Takada, T., Takahashi, F., Takahashi, T., & Osawa, K. (2019). Gambling symptoms, behaviors, and cognitive distortions in Japanese university students. *Substance Abuse: Treatment Prevention and Policy*, *14*(51), 1–7. 10.1186/s13011-019-0230-5 31722743 10.1186/s13011-019-0230-5 PMC6854769

